# Cytogenetic data on the agro-predatory ant *Megalomyrmex incisus* Smith, 1947 and its host, *Mycetophylax conformis* (Mayr, 1884) (Hymenoptera, Formicidae)

**DOI:** 10.3897/CompCytogen.v11i1.10842

**Published:** 2017-01-09

**Authors:** Danon Clemes Cardoso, Tássia Tatiane Pontes Pereira, Alessandro Lick Cordeiro, Maykon Passos Cristiano

**Affiliations:** 1 Departamento de Biodiversidade, Evolução e Meio Ambiente – Universidade Federal de Ouro Preto, Ouro Preto, Brazil; 2 Laboratório de Genética Evolutiva e de Populações – Universidade Federal de Ouro Preto, Ouro Preto, Brazil; 3 Programa de Pós-graduação em Ecologia – Universidade Federal de Viçosa, Viçosa, Brazil; 4 Departamento de Genética – Universidade Federal do Paraná, Curitiba, Brazil

**Keywords:** Karyotype, chromosome counts, ants, biodiversity, evolution

## Abstract

We provide the first karyotype description of the agro-predatory ant species *Megalomyrmex incisus* Smith, 1947 (Myrmicinae, Formicidae), and chromosome counts of its host *Mycetophylax conformis* (Mayr, 1884) (Myrmicinae, Formicidae) from geographically distinct populations. Colonies of both species were sampled from coastal areas of Ilhéus, Bahia, Brazil, and transferred to the laboratory. Metaphase spreads were prepared from the cerebral ganglia of defecated larvae. The slides were examined and pictures of the best metaphases were taken. The chromosome number for *Megalomyrmex incisus* was 2n=50 and n=25. The karyotype of this species consists of 20 metacentric and 5 submetacentric pairs. Thus, the karyotype formula of the diploid set was 2K=40M + 10SM and a fundamental number FN=100. The host species *Mycetophylax conformis* has 2n=30 and the karyotype consisting of 11 metacentric and 4 submetacentric pairs. The karyotype formula was 2K=22M + 8SM, and a fundamental number FN=60. *Megalomyrmex incisus* showed a slightly higher chromosome number, placed at the marginal range of the known distribution of haploid karyotypes of the Myrmicinae. The chromosome number and chromosomal morphology of *Mycetophylax conformis* corresponded to those of previously studied populations, suggesting its karyotype stability.

## Introduction

Chromosomes are the units of inheritance bearing the complete set of information necessary for biological development. In general, species have a fixed number of chromosomes, and closely related species tend to have more similar karyotypes than distantly related ones (Guerra 2013). Changes in karyotype features (e.g., chromosome number and morphology) may have evolved through multiple speciation events, each involving the fixation of particular chromosomal rearrangements (Schubert and Lysak 2011).

Ants are among the insect taxa that exhibit one of the most variable chromosome numbers, ranging from n = 1 to 60 (reviewed by Lorite and Palomeque 2010). This high karyotype diversity seems to be correlated to ant diversification, which currently comprises nearly 14,000 described species in 21 subfamilies (Agosti and Johnson 2016). Cytogenetic data are available for about 750 ant species (Lorite and Palomeque 2010), with more data accumulating rapidly; this information has advanced our understanding of ant systematics and evolution (Mariano et al. 2012, Cristiano et al. 2013, Cardoso et al. 2014). However, with the growing number of ant species, more cytogenetic studies are needed to reveal the extent of chromosome diversity.

Myrmicinae is the most diverse subfamily of Formicidae, and it consequently encompasses the majority of species with described karyotypes (Lorite and Palomeque 2010). However, karyotype information is not yet available for some widely distributed genera of this subfamily. For example, *Megalomyrmex* Forel, 1885 comprises 44 described species distributed from Mexico to northern Argentina (Longino 2010), and despite this broad geographic occurrence, cytogenetic data are nonexistent. The genus contains social parasites of fungus-growing ants, with *Megalomyrmex incisus* Smith, 1947 recently described as an agro-predatory ant of *Mycetophylax* Emery, 1913 species (Cardoso et al. 2016). Here, we describe for the first time the karyotype of the agro-predator *Megalomyrmex incisus* as well as its host, the fungus-growing ant *Mycetophylax conformis* (Mayr, 1884).

## Material and methods

### Colony sampling

Colonies of both species were sampled on the coast of Ilhéus, Bahia, Brazil (14°47'36.61"S, 39°2'46.96"W). A colony of *Megalomyrmex incisus* was collected during excavation of the colony of *Mycetophylax conformis* (see Cardoso et al. 2016). The entrances to the colony are located on the top of a small sand turret and surrounded by a sand crater. Excavation was carried out according to protocols developed by Cardoso et al. (2011). Thirteen colonies in total were excavated, and *Megalomyrmex incisus* were collected from one putative nest of *Mycetophylax conformis*. Colonies were collected in their entirety and transported to the laboratory, where they were transferred to rearing systems as described by Cardoso et al. (2011). Species were kept under laboratory conditions (9:15 L:D photoperiod, 25 °C) in order to obtain broods for performing cytogenetic analysis.

### Karyotype descriptions

*Mycetophylax conformis* was karyotyped to determine whether it has the same chromosome number as populations characterized by Cardoso et al. (2014), as divergent chromosome numbers were already found in some congeneric species. Larvae obtained from the colonies maintained in the laboratory were used for karyotype characterization. One colony of *Megalomyrmex incisus* and eleven out of thirteen colonies of *Mycetophylax conformis* were evaluated cytogenetically. For *Megalomyrmex incisus* 30 individuals were used in cytogenetic analyses, whereas for *Mycetophylax conformis* the numbers of individuals analyzed per colony were: ten individuals in five colonies; 8 individuals in two colonies; 6 individuals in three colonies and 4 individuals in one colony. Metaphase spreads were prepared from the cerebral ganglia of prepupae, according to Imai et al. (1988). The cerebral ganglion was dissected in colchicine–hypotonic solution (0.005% w/v colchicine in 1% sodium citrate solution) under a stereoscopic microscope, transferred to a new drop of colchicine–hypotonic solution and incubated in the dark for one hour (see Imai et al. 1988 and Cardoso et al. 2012 for the detailed procedure). The slides were evaluated using a phase contrast microscope. Quality metaphase slides were stained with 4% Giemsa solution in Sørensen’s buffer (pH 6.8); the best metaphases were photographed using an Olympus BX51 microscope equipped with a digital camera, and then used for evaluation of the chromosome number and morphology. Chromosomes were classified following the nomenclature proposed by Levan et al. (1964), which is based on centromere positions: acrocentric (A), subtelocentric (ST), submetacentric (SM), and metacentric (M). We measured ten (*Mycetophylax conformis*) and seven (*Megalomyrmex incisus*) spread metaphases with chromosomal integrity, evident centromeres, and without overlapping during the morphometric karyotype analysis. The following features of chromosomes were evaluated: total length (TL), long arm length (L), short arm length (S), arm ratio between the long and short arms (r = L/S), relative chromosome length (RL) of each chromosome (TL ×100/∑TL) and asymmetry index (∑long arms/∑total length ×100). In order to identify putative cytogenetic markers in *Megalomyrmex incisus*, sequential fluorochrome staining with chromomycin A_3_/distamycin A/4’, 6–diamidino–2–phenylindole (CMA_3_/DA/DAPI) was done according to Schweizer (1980) to characterize CG- and AT-rich regions. These slides were analyzed under an epifluorescence microscope (Zeiss AxioImager Z2) equipped with a digital camera (AxioCam MRc). The fluorescence signals were analyzed using two different filters: a GFP filter (450 to 480 nm) for CMA_3_, and a DAPI filter (330 to 385 nm) for DAPI.

## Results

**Figure 1.**
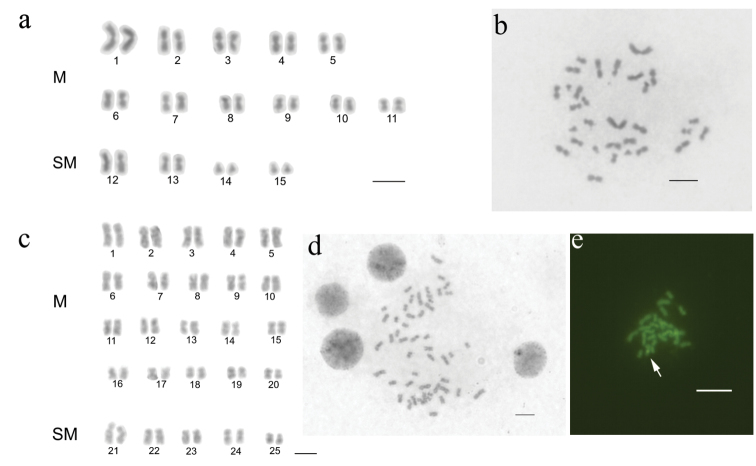
Cytogenetic data of *Megalomyrmex incisus* and its host *Mycetophylax conformis*. **a** *Mycetophylax conformis* conventional staining of diploid karyotype and **b** metaphase **c** *Megalomyrmex incisus* conventional staining of diploid karyotype and **d** metaphase **e** *Megalomyrmex incisus* metaphase stained with CMA_3_, white arrow indicates positive staining for CMA_3_. M=metacentric, SM=submetacentric. Bar = 5 µm.

All individuals of *Mycetophylax conformis* from Ilhéus had chromosome counts of 2n=30 (Fig. 1a–b). The karyotype of this species consists of 11 metacentric (M) pairs and 4 submetacentric (SM) pairs ranging in size from large to small. The mean total length of individual chromosomes ranged from 5.49 to 1.59 µM, while the mean total length of all chromosomes was 90.18 µM. The karyotypic formula of the diploid set was 2K=22M + 8SM. Thus, a fundamental number (number of chromosome arms in the diploid karyotype) was FN=60. Morphometric data for chromosomes of *Mycetophylax conformis* are shown in Table 1.

**Table 1.** Detailed karyotype analysis of *Mycetophylax conformis*.

Chromosome	TL (µM)	L (µM)	S (µM)	RL	*r*	Chromosome classification
**1**	5.49±0.60	2.99±0.30	2.5±0.32	6.09±0.29	1.19±0.07	Metacentric
**1**	5.3±0.52	2.9±0.29	2.41±0.25	5.88±0.32	1.19±0.06	Metacentric
**2**	4.08±0.38	2.18±0.23	1.9±0.17	4.53±0.23	1.16±0.07	Metacentric
**2**	3.96±0.40	2.06±0.14	1.9±0.26	4.39±0.20	1.09±0.08	Metacentric
**3**	3.64±0.40	2.01±0.30	1.63±0.15	4.03±0.12	1.22±0.17	Metacentric
**3**	3.54±0.32	1.95±0.15	1.59±0.19	3.92±0.13	1.22±0.11	Metacentric
**4**	3.44±0.36	1.83±0.21	1.61±0.23	3.81±0.11	1.09±0.19	Metacentric
**4**	3.36±0.37	1.82±0.17	1.54±0.24	3.72±0.10	1.19±0.16	Metacentric
**5**	3.25±0.29	1.74±0.19	1.51±0.14	3.61±0.09	1.13±0.12	Metacentric
**5**	3.19±0.28	1.75±0.10	1.44±0.24	3.54±0.07	1.17±0.22	Metacentric
**6**	3.08±0.28	1.7±0.15	1.38±0.19	3.41±0.10	1.24±0.18	Metacentric
**6**	3.04±0.26	1.66±0.22	1.38±0.16	3.37±0.07	1.1±0.23	Metacentric
**7**	2.96±0.25	1.64±0.10	1.32±0.21	3.29±0.13	1.25±0.22	Metacentric
**7**	2.92±0.27	1.73±0.16	1.2±0.12	3.24±0.15	1.47±0.10	Metacentric
**8**	2.85±0.28	1.6±0.22	1.25±0.12	3.16±0.13	1.3±0.18	Metacentric
**8**	2.78±0.27	1.57±0.12	1.21±0.17	3.08±0.10	1.34±0.11	Metacentric
**9**	2.63±0.37	1.56±0.24	1.08±0.15	2.91±0.19	1.42±0.16	Metacentric
**9**	2.45±0.30	1.38±0.15	1.07±0.18	2.71±0.13	1.31±0.17	Metacentric
**10**	2.29±0.23	1.29±0.13	1±0.16	2.54±0.15	1.32±0.22	Metacentric
**10**	2.23±0.18	1.24±0.17	0.99±0.09	2.48±0.13	1.3±0.21	Metacentric
**11**	2.41±0.64	1.4±0.43	1.01±0.22	2.68±0.71	1.31±0.19	Metacentric
**11**	2.3±0.60	1.28±0.41	1.01±0.22	2.55±0.67	1.15±0.25	Metacentric
**12**	3.65±0.75	2.36±0.48	1.29±0.28	4.03±0.64	1.78±0.12	Submetacentric
**12**	3.52±0.72	2.32±0.41	1.2±0.32	3.89±0.58	1.98±0.23	Submetacentric
**13**	2.57±0.16	1.65±0.14	1±0.25	2.87±0.36	1.86±0.21	Submetacentric
**13**	2.49±0.13	1.59±0.10	0.93±0.26	2.78±0.27	2.02±0.22	Submetacentric
**14**	1.78±0.10	1.33±0.29	0.77±0.56	1.98±0.10	2.32±0.30	Submetacentric
**14**	1.75±0.15	1.3±0.27	0.73±0.55	1.94±0.10	2.42±0.38	Submetacentric
**15**	1.65±0.14	1.25±0.36	0.75±0.60	1.83±0.14	2.18±0.35	Submetacentric
**15**	1.59±0.13	1.28±0.41	0.77±0.62	1.77±0.15	2.22±0.30	Submetacentric
∑	90.18					

TL (µM) total chromosome length in micrometers; L (µM) long arm length in micrometers; S (µM) short arm length in micrometers; RL relative chromosome length; *r* arm ratio (*r*=L/S).

The chromosome number for *Megalomyrmex incisus* was 2n=50 and n=25 (Fig. 1c–e). The karyotype of this species consists of 20 metacentric (M) pairs and 5 submetacentric (SM) pairs, with less variation in size (Fig. 1c). The mean length of individual chromosomes ranged from 4.65 to 1.85 µM, while the mean total length of all chromosomes was 141.89 µM (Table 2). The karyotypic formula of the diploid set was 2K=40M + 10SM, and a fundamental number was FN=100. Only haploid (male) brood was subjected to sequential fluorochrome staining, which revealed positive GC-rich blocks (CMA3+) in a single chromosome at the pericentromeric region (Fig. 1e). AT-rich blocks were not found since the chromosomes were stained uniformly (data not shown).

**Table 2.** Detailed karyotype analysis of *Megalomyrmex incisus*.

Chromosome	TL (µM)	L (µM)	S (µM)	RL	*r*	Chromosome classification
**1**	4.65±0.92	2.56±0.53	2.09±0.41	3.28±0.20	1.19±0.092	Metacentric
**1**	4.25±0.85	2.58±0.49	1.71±0.42	3.00±0.10	1.32±0.201	Metacentric
**2**	4.2±0.84	2.36±0.42	1.84±0.45	2.96±0.13	1.33±0.175	Metacentric
**2**	4.15±0.76	2.25±0.52	1.88±0.26	2.92±0.13	1.2±0.150	Metacentric
**3**	3.84±0.80	1.97±0.58	1.88±0.25	2.71±0.08	1.09±0.192	Metacentric
**3**	3.68±0.85	2.04±0.49	1.63±0.40	2.59±0.13	1.25±0.129	Metacentric
**4**	3.46±0.83	1.99±0.50	1.6±0.35	2.44±0.11	1.26±0.136	Metacentric
**4**	3.39±0.82	1.9±0.58	1.61±0.32	2.39±0.12	1.17±0.278	Metacentric
**5**	3.37±0.80	1.83±0.44	1.55±0.37	2.38±0.12	1.21±0.087	Metacentric
**5**	3.25±0.75	1.89±0.42	1.36±0.36	2.29±0.11	1.31±0.123	Metacentric
**6**	3.1±0.74	1.72±0.33	1.43±0.42	2.18±0.11	1.13±0.130	Metacentric
**6**	3.09±0.69	1.75±0.36	1.35±0.32	2.18±0.08	1.24±0.071	Metacentric
**7**	3.05±0.69	1.73±0.39	1.41±0.33	2.15±0.09	1.28±0.183	Metacentric
**7**	3.02±0.67	1.75±0.37	1.36±0.35	2.13±0.08	1.21±0.231	Metacentric
**8**	2.98±0.67	1.6±0.34	1.39±0.33	2.10±0.08	1.17±0.047	Metacentric
**8**	2.91±0.65	1.58±0.36	1.33±0.31	2.05±0.07	1.21±0.100	Metacentric
**9**	2.86±0.62	1.55±0.33	1.34±0.30	2.01±0.05	1.17±0.085	Metacentric
**9**	2.81±0.63	1.6±0.38	1.29±0.30	1.98±0.06	1.16±0.191	Metacentric
**10**	2.8±0.59	1.55±0.43	1.29±0.18	1.97±0.04	1.28±0.174	Metacentric
**10**	2.72±0.60	1.43±0.31	1.3±0.29	1.92±0.05	1.15±0.065	Metacentric
**11**	2.64±0.60	1.51±0.43	1.2±0.18	1.86±0.07	1.33±0.174	Metacentric
**11**	2.58±0.62	1.48±0.31	1.2±0.33	1.81±0.09	1.17±0.205	Metacentric
**12**	2.49±0.64	1.45±0.38	1.18±0.30	1.75±0.11	1.18±0.219	Metacentric
**12**	2.47±0.61	1.4±0.25	1.12±0.38	1.74±0.10	1.24±0.204	Metacentric
**13**	2.46±0.57	1.28±0.31	1.22±0.27	1.73±0.08	1.14±0.122	Metacentric
**13**	2.42±0.55	1.35±0.28	1.13±0.27	1.71±0.07	1.18±0.089	Metacentric
**14**	2.4±0.52	1.26±0.33	1.18±0.23	1.69±0.07	1.12±0.177	Metacentric
**14**	2.37±0.50	1.27±0.25	1.12±0.26	1.67±0.07	1.15±0.080	Metacentric
**15**	2.37±0.50	1.32±0.35	1.12±0.18	1.67±0.08	1.17±0.147	Metacentric
**15**	2.32±0.49	1.33±0.23	1.06±0.28	1.63±0.06	1.13±0.153	Metacentric
**16**	2.26±0.48	1.34±0.22	1.04±0.30	1.59±0.08	1.1±0.262	Metacentric
**16**	2.19±0.48	1.23±0.31	0.97±0.20	1.55±0.08	1.19±0.164	Metacentric
**17**	2.07±0.53	1.23±0.32	0.89±0.24	1.46±0.11	1.44±0.186	Metacentric
**17**	2.02±0.43	1.13±0.24	0.92±0.20	1.42±0.07	1.24±0.164	Metacentric
**18**	2±0.37	1.11±0.17	0.89±0.21	1.41±0.06	1.24±0.117	Metacentric
**18**	1.94±0.36	1.12±0.20	0.85±0.17	1.37±0.06	1.27±0.120	Metacentric
**19**	1.88±0.33	1.03±0.19	0.89±0.15	1.33±0.06	1.16±0.097	Metacentric
**19**	1.85±0.33	1.09±0.13	0.79±0.22	1.30±0.05	1.26±0.176	Metacentric
**20**	2.05±0.94	1.13±0.70	0.89±0.25	1.44±0.63	1.31±0.343	Metacentric
**20**	1.98±0.79	1.09±0.64	0.86±0.16	1.39±0.53	1.25±0.392	Metacentric
**21**	3.45±1.14	2.2±0.86	1.24±0.29	2.43±0.54	1.79±0.321	Submetacentric
**21**	3.35±0.97	2.18±0.70	1.15±0.30	2.36±0.49	1.76±0.369	Submetacentric
**22**	3.4±0.37	2.29±0.21	1.13±0.20	2.40±0.35	1.93±0.248	Submetacentric
**22**	3.13±0.37	1.96±0.36	1.06±0.51	2.21±0.27	1.82±0.292	Submetacentric
**23**	3±0.37	1.91±0.26	1.02±0.18	2.12±0.22	1.87±0.293	Submetacentric
**23**	2.9±0.27	1.86±0.21	1.01±0.10	2.04±0.21	1.83±0.174	Submetacentric
**24**	2.8±0.28	1.78±0.17	1.02±0.16	1.98±0.21	1.74±0.270	Submetacentric
**24**	2.62±0.31	1.66±0.26	0.96±0.06	1.86±0.24	1.74±0.174	Submetacentric
**25**	2.5±0.25	1.64±0.18	0.85±0.09	1.76±0.28	1.89±0.150	Submetacentric
**25**	2.39±0.22	1.57±0.09	0.82±0.14	1.69±0.27	1.91±0.263	Submetacentric
∑	141.89					

TL (µM) total chromosome length in micrometers; L (µM) long arm length in micrometers; S (µM) short arm length in micrometers; RL relative chromosome length; *r* arm ratio (*r*=L/S).

## Discussion

Four distinct karyotypes have been reported for *Mycetophylax*, from three valid species: *Mycetophylax morschi* (Emery, 1888) harbors two cytotypes, 2n=26 and 2n=30, whereas *Mycetophylax simplex* (Emery, 1888) and *Mycetophylax conformis* harbor 2n=36 and 2n=30, respectively (Cardoso et al. 2014). Here we evaluated the chromosome counts of *Mycetophylax conformis* from Bahia, Brazil, the host of the social parasite *Megalomyrmex incisus* (Cardoso et al. 2016). The studied population is located 1,000 km from the northern population that was cytogenetically analyzed by Cardoso et al. (2014). The diploid number of chromosomes and their morphology did not differ from previously characterized populations, showing 2n=30 with the karyotype comprising metacentric and submetacentric chromosomes. These results suggest that *Mycetophylax conformis* has a stable karyotype.

Karyotype variation among populations can occur across different species (Lukhtanov et al. 2011). Populations on the edge of distribution ranges of various species were reported to have generally adaptive chromosomal variations (Singh 2015). Clinal variation in ant chromosome structure has also been observed in *Typhlomyrmex rogenhoferi* Mayr, 1862, with populations from Bahia and Pará in Brazil and French Guiana (each 1,000 km apart) showed diploid chromosome numbers of 38, 34 and 36, respectively (Mariano et al. 2006). The population of *Mycetophylax conformis* studied in the present paper was sampled about 900 km northeast of the population evaluated by Cardoso et al. (2014) in Rio de Janeiro State. It is known that great distances between populations can promote conspicuous genetic variation; however, chromosome counts and karyotype structure of *Mycetophylax conformis* were identical across the whole distribution range, supporting the proposed chromosomal stability of the species.

*Megalomyrmex incisus* is the first cytogenetically characterized species of *Megalomyrmex*. It showed a chromosome number of 2n=50 (the haploid number was n=25) with 20 metacentric pairs and five submetacentric pairs. The chromosome number of *Megalomyrmex incisus* is consistent with the range of karyotypic variation in the Formicidae (n=1 to 60; reviewed by Lorite and Palomeque 2010). However, this particular number is rare in the subfamily Myrmicinae, and it thus represents the marginal distribution frequency of chromosome numbers known for this group (Lorite and Palomeque 2010).

The fluorochrome staining confirms a cytological marker that was identified in a number of ant species (Mariano and Delabie 2013), as well in other insects (Cardoso et al. 2012). A single chromosome in haploid males showed a pericentromeric positive GC-rich block (CMA_3_^+^). Indeed, different ant species show only one pair of chromosomes in diploid females that typically bears a positive GC-rich block. However, these positive GC-rich blocks may differ in the location on the chromosome. For instance, in *Mycetophylax conformis* the CMA_3_^+^ block is in the telomeric region, while in *Mycetophylax simplex* it is pericentromeric (Cardoso et al. 2014). Previous studies have shown that these positive CG-rich blocks are correlated with the location of nucleolus organizer regions (NORs) and rDNA sites (Cardoso et al. 2012; Barros et al. 2015), indicating that they may represent NORs in this particular case as well.

An accurate karyotype description should take into account physical measurements like length of individual chromosomes, total karyotype length, and arm length ratios. These types of features allow accurate identification of chromosomes, which is critical for robust karyotype analysis. Morphometric data on ant chromosomes are still scarce and exist for only a few species (e.g. Cristiano et al. 2013; Barros et al. 2014), limiting our ability to further extrapolate evolutionary patterns or trajectories. The measurements of chromosomes presented here for *Megalomyrmex incisus* and *Mycetophylax conformis* allow accurate descriptions of chromosome morphology. As more data became available, general karyotypic patterns can be revealed, increasing our general understanding of chromosome evolution in ants.
